# *TUSC7* expression and mutational profile define its potential as a diagnostic and therapeutic biomarker in non-small cell lung cancer

**DOI:** 10.1186/s40364-026-00916-0

**Published:** 2026-04-10

**Authors:** Pablo Martin-Lopez, Marta Cuadros, Fernando Montenegro-Elvira, Alberto M. Arenas, María Palomino, Claudia Loidi, Mónica Saiz, Pedro P. Medina

**Affiliations:** 1 Gene Expression Regulation and Cancer Group (CTS-993), GENYO, Centre for Genomics and Oncological Research: Pfizer-University of Granada-Andalusian Regional Government, Granada, Spain; 2 Department of Biochemistry and Molecular Biology I, Faculty of Sciences, University of Granada, Avda. De Fuentenueva S/N, Granada, 18071 Spain; 3 Instituto Biosanitario de Granada (Ibs.Granada), Granada, Spain; 4 Pathological Anatomy, Universitary Hospital Cruces, University of Pais Vasco, Gipuzkoa, Spain; 5 Department of Biochemistry and Molecular Biology III and Immunology, University of Granada, Granada, Spain; 6 Present Address: Department of Oncology-Pathology, Karolinska Institute, Stockholm, Sweden

**Keywords:** Non-small cell lung cancer, Variant, Long non-coding RNA, Biomarker, Diagnostic

## Abstract

Non-small cell lung cancer (NSCLC) accounts for ~85% of lung cancer cases and remains the leading cause of cancer-related mortality worldwide. Despite advances in targeted therapies, early diagnosis and prediction of treatment response remain major challenges. Long non-coding RNAs (lncRNAs) are increasingly recognized as tissue-specific regulators of gene expression with growing relevance in cancer biology, making them attractive candidates for biomarker discovery. To identify functionally relevant lncRNA variants in NSCLC, we analyzed targeted DNA sequencing data from 39 NSCLC-derived cell lines and 70 primary tumors, including matched adjacent non-tumoral tissues to enable somatic variant identification. A somatic variant in the lncRNA *TUSC7* was ranked among the top candidates, supported by high functional impact scores. Located in exon 4, a region previously reported as functionally relevant, the mutation was predicted to alter RNA secondary structure and was experimentally associated with enhanced cell fitness under genotoxic stress. *TUSC7* variant altered lncRNA stability under genotoxic stress, supporting a possible *TUSC7* DNA damage-related role. In addition, in our independent cohort, *TUSC7* expression was significantly downregulated in tumor samples compared with matched adjacent non-tumoral tissue, a pattern that was independently validated using transcriptomic data from the Clinical Proteomic Tumor Analysis Consortium (CPTAC). Receiver operating characteristic (ROC) analysis yielded AUC values above 0.96 in both datasets, underscoring the strong diagnostic potential of *TUSC7* expression in NSCLC. Together, these findings position *TUSC7* as a promising biomarker for tumor detection and a potential candidate for therapeutic stratification.

**Supplementary Information:**

The online version contains supplementary material available at 10.1186/s40364-026-00916-0.

**To the editor**: NSCLC accounts for approximately 85% of lung cancer cases [1], and remains the leading cause of cancer-related mortality worldwide [2]. This highlights the need for improved strategies in diagnosis, prognosis and treatment. Non-coding RNAs have recently attracted considerable attention due to their involvement in carcinogenesis [3] and their potential as biomarkers [4]. Among them, lncRNAs, defined as transcripts longer than 200 nucleotides that are not translated into proteins, have arisen as potentially relevant players in cancer biology [4]. LncRNAs have emerged as tissue-specific key molecules that can intervene in several cancer hallmarks [5], underscoring their potential as diagnostic and prognostic biomarkers [4].

Here we aimed to identify high-impact lncRNA mutations in NSCLC by performing targeted DNA-sequencing on 39 NSCLC-derived cell lines and 70 primary tumors, including matched adjacent non-tumoral tissues that were used to identify somatic variants (Fig. 1a). The panel included 2481 exonic regions of 827 lncRNAs (Additional Files 1 and 2), and 1423 somatic mutations were identified across 524 of these genes (Additional File 3). Functional impact of the mutations was assessed using CADD and FATHMM-MKL [6, 7] (Additional File 4). After applying stringent thresholds (CADD PHRED score > 20 and FATHMM-MKL non-coding score > 0.98), we obtained a shortlist of 17 candidate mutations (Additional File 5). Among the mutated lncRNAs, two (*TUSC7* and *SOX2-OT*) harboured more than 10 mutations in our cohort. For experimental follow-up, we prioritized the mutation with the highest CADD score between these two, which corresponded to a somatic variant in *TUSC7* (chr3:116716439 *T* > A; c.1510T > A in transcript NR_015391.1). *Tumor Suppressor Candidate 7* (*TUSC7*) is a long non-coding RNA initially identified as a tumor suppressor in osteosarcoma and later implicated in colon and other tumor types [8]. Interestingly, the c.1510T > A mutation we detected lies within exon 4, previously reported as functionally relevant in *TUSC7* biology [8]. RNAfold predictions indicate that the single nucleotide variant (SNV) induces a conformational change in the transcript’s secondary structure (Fig. 1b), supporting a potential structural impact of the mutation [9]. Sanger sequencing confirmed the mutant’s presence in both genomic DNA (gDNA) and complementary DNA (cDNA) exclusively in the tumor sample (Fig. 1c), supporting its classification as a somatic, cancer-specific mutation.

**Fig. 1:**
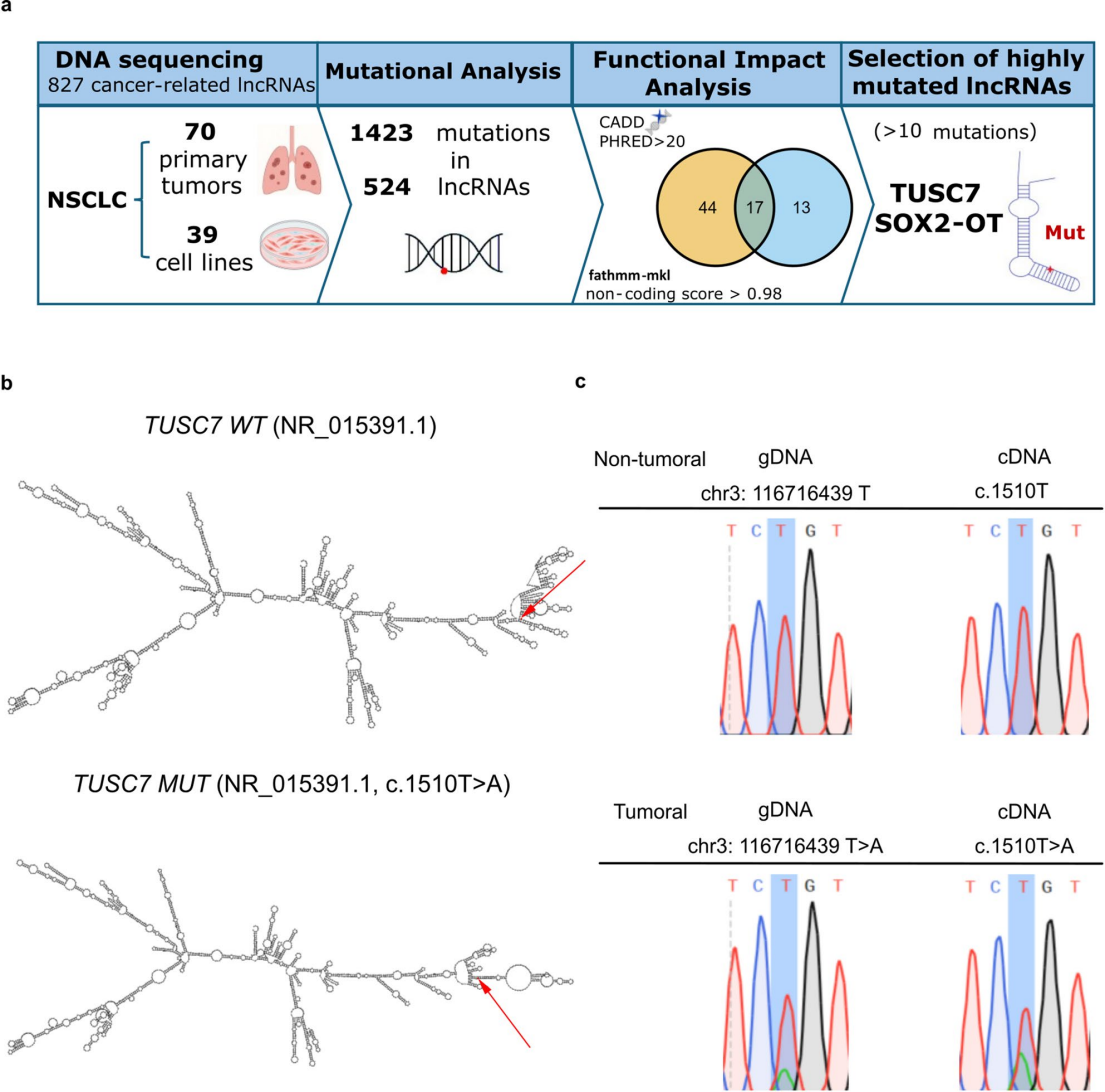
High-impact variant identification in *TUSC7*. (**a**) Strategy for the identification of functionally relevant lncRNA mutations in NSCLC. Targeted sequencing of 827 cancer-related lncRNAs (2481 exonic regions) was performed in 70 primary tumors and 39 NSCLC-derived cell lines. Mutational analysis identified 1423 somatic mutations across 524 lncRNAs. Functional prioritization using CADD (PHRED > 20) and FATHMM-MKL (non-coding score > 0.98) yielded 17 high-impact candidate mutations. Among lncRNAs harboring > 10, *TUSC7* and *SOX2-OT* were selected for follow-up, with *TUSC7* prioritized due to its highest predicted functional impact score. (**b**) RNA secondary structure prediction of both *TUSC7* wild-type (*TUSC7 WT*) and mutant TUSC7 (*TUSC7 MUT*) according to the minimum free energy (MFE) fold algorithm. Red arrows indicate the position of the c.1510T > A variant. (**c**) Sanger sequencing results of *TUSC7* region containing the identified variant in both tumoral (bottom panels) and matched non-tumoral samples (upper panels). The presence of the SNV was assessed at genomic DNA (gDNA, left panels) and complementary DNA (cDNA, right panels) levels

To validate our sequencing findings and in silico functional predictions, we identified the NSCLC H460 cell line for functional assays, as it harbors a genomic profile consistent with a homozygous deletion of the *TUSC7* locus. This was confirmed by the absence of PCR amplification of exons 1 and 4 and undetectable expression by qPCR (Supp. Fig. 1a, b; Supp. Fig 4a). H460 therefore provided a suitable model to reintroduce either wild-type *TUSC7* (*TUSC7 WT*) or mutant *(TUSC7 MUT)*. H460 cells were transduced with *TUSC7 WT*, *TUSC7 MUT*, or empty vector (EV) control (Supp. Fig. 2a). Comparable expression levels between *WT* and *MUT* constructs were verified by FACS and qPCR (Supp. Fig. 2a and b), ensuring that downstream phenotypic differences were not attributable to differential expression levels.

Under basal conditions, *TUSC7* overexpression did not significantly affect cell fitness, colony formation, viability, proliferation or migration (Supp. Fig. 3a–f). Given that *TUSC7* is a p53-regulated lncRNA [8], we further assessed responses to DNA damage using doxorubicin and mitomycin C (Fig. 2a; Supp. Fig. 3c–f). Under doxorubicin-induced genotoxic stress, *TUSC7* *MUT* expression was associated with a competitive growth advantage and increased viability at day 7 (Fig. 2a; Supp. Fig. 3c), whereas no differences were observed under basal conditions. This stress-dependent phenotype was also reproduced in additional NSCLC models (H1299 and SK-MES-1) (Fig. 2b; Supp. Fig. 2c–f). To further strengthen the translational relevance of these findings, we tested whether this phenotype could be extended to NSCLC clinically relevant DNA-damaging agents, such as cisplatin. In lung squamous cell carcinoma (LUSC) SK-MES-1 cells, cisplatin dose-response assays demonstrated reduced sensitivity of the *MUT* variant relative to *WT*, as reflected by higher IC_50_ values (Fig. 2c). Actinomycin D assays in H460 cells further showed decreased stability of the *MUT* transcript under genotoxic stress but not basal conditions (Fig. 2d), indicating that altered transcript stability may partially contribute to the observed stress-dependent phenotype. These results are in line with previous reports linking low *TUSC7* expression to genotoxic resistance in glioblastoma [10], and support further investigation of this mutation as a candidate biomarker of therapy response in NSCLC.

**Fig. 2:**
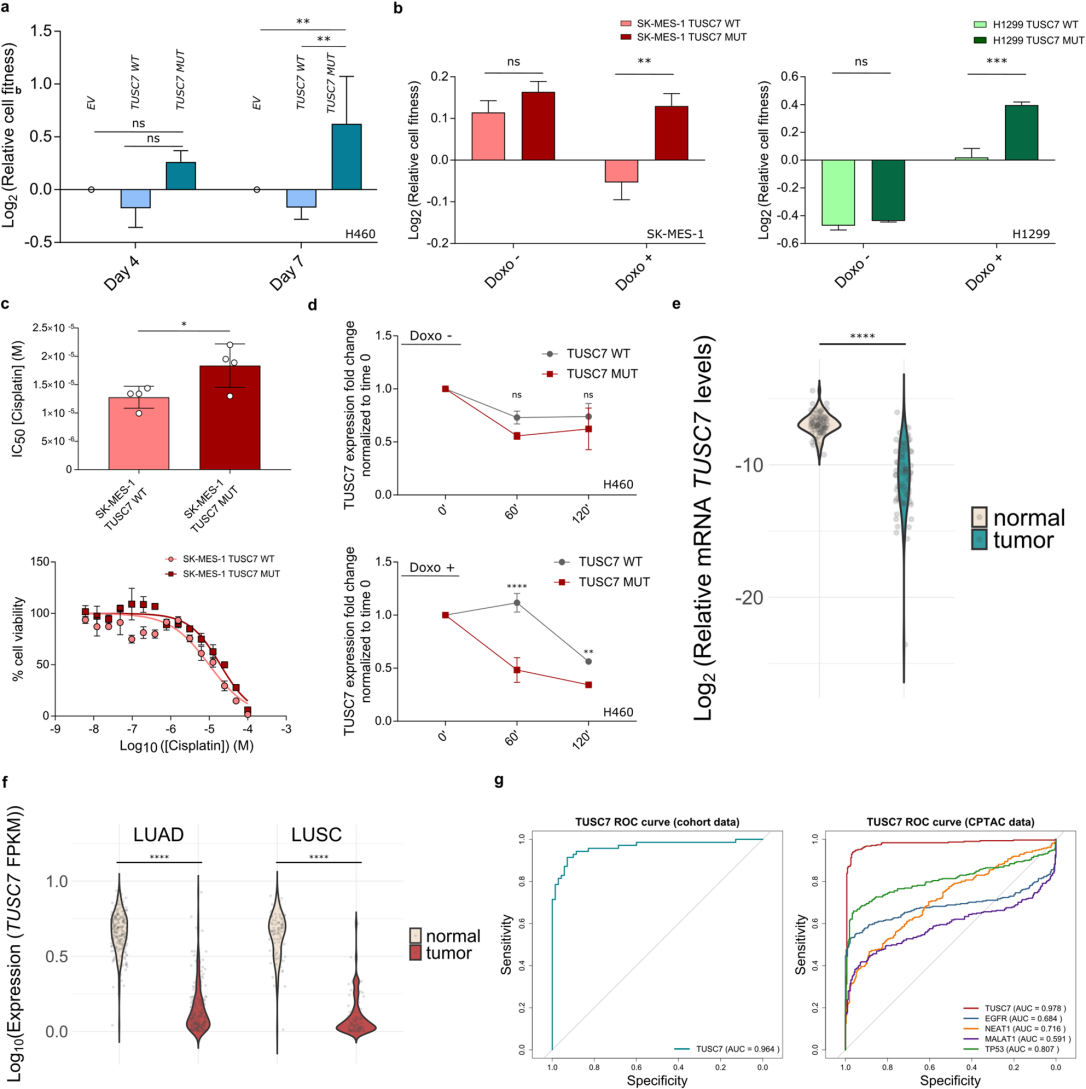
TUSC7 expression and variants as biomarkers in therapeutics and tumor detection. **(a)** Competitive assay under genotoxic conditions in H460 cell line after transduction with lentiviral particles carrying an empty vector (EV), wild-type (*WT*) *TUSC7* or mutant (*MUT*) *TUSC7*. The different conditions were normalized to day 0 and EV control. Three independent biological replicates were performed. (Day 7 H460 *TUSC7 WT* vs H460 *TUSC7 MUT*: p = 0.014, mean diff. = −0.80 (95% confidence interval (CI) −1.24 – −0.34); Day 7 H460 EV vs H460 *TUSC7 MUT*: p = 0.008, mean diff. = −0.62 (95% CI −1.08 – −0.17)). **(b)** Day 7 competitive assays results under basal (Doxo−) and genotoxic (Doxo+) conditions in NSCLC SK-MES-1 (left panel) and H1299 (right panel) cell lines transduced with either *TUSC7 WT* or *TUSC7 MUT*. Results are normalized to day 0 for each condition. Three biological replicates were performed. (SK-MES-1 *TUSC7 WT* vs SK-MES-1 *TUSC7 MUT*: p = 0.0034, mean diff. = 0.18 (95% CI 0.10–0.27); H1299* TUSC7 WT* vs H1299 *TUSC7 MUT*: p = 0.0007, mean diff. = 0.38 (95% CI 0.27–0.49)). **(c)** SK-MES-1 *TUSC7 WT* and SK-MES-1 *TUSC7 MUT* average IC_50_ values (upper panel) and representative dose-response curves (bottom panel). Four biological replicates were performed. (SK-MES-1 *TUSC7 WT* IC_50_ vs SK-MES-1 *TUSC7 MUT* IC_50_: p = 0.0409, mean diff. = 5.58 × 10−6 (95% CI 3.19 × 10−7–1.08 × 10−5). **(d)** Actinomycin D experiments performed in NCI-H460 cell line transduced with either *TUSC7 WT* or *TUSC7 MUT* variant under basal (upper panel) or genotoxic stress (bottom panel) conditions. Three biological replicates were performed. (60’ Doxo+ H460 *TUSC7 WT* vs H460 *TUSC7 MUT*: p < 0.0001, mean diff. = 0.63 (95% CI 0.50–0.77); 120’ Doxo+ H460 *TUSC7 WT* vs H460 *TUSC7 MUT*: p = 0.0022, mean diff. = 0.22 (95% CI 0.08–0.36)). **(e)** Violin plot representing the distribution of *TUSC7* mRNA levels in tumor and non-tumor paired samples obtained from internal patient cohort. **(f)** Violin plot representing the distribution of *TUSC7* mRNA levels in paired tumor and non-tumor samples obtained from both LUAD and LUSC CPTAC cohorts. **(g)** Left panel: receiver operating characteristic (ROC) curve and area under the curve (AUC) values for *TUSC7* expression in internal LUAD cohort (95% CI 0.93–0.99). Right panel: ROC and AUC values for *TUSC7* (95% CI 0.97–0.99), *NEAT1*, *MALAT1*, *EGFR*, and *TP53* expression in CPTAC NSCLC cohort (LUAD, n = 215; LUSC, n = 96). ns: not significant; * p < 0.05; ** p < 0.01; *** p < 0.001; **** p < 0.0001. FPKM: fragments per kilobase of transcript per million mapped reads

Consistent with prior reports describing reduced *TUSC7* expression in NSCLC tissues [11], we evaluated its diagnostic potential. In our cohort (*n* = 70) and paired tumor-healthy tissues from CPTAC data (lung adenocarcinoma (LUAD), *n* = 215; LUSC, *n* = 96), *TUSC7* expression was significantly lower in tumors (Fig. 2e–f). Generalized linear mixed-effects model (GLMM)-based ROC analyses yielded AUC values of 0.9639 (internal cohort) and 0.9781 (CPTAC) (Fig. 2g), indicating strong discrimination in distinguishing tumor from non-tumoral tissue. Comparative analyses suggested that *TUSC7* performs favorably relative to selected NSCLC-associated genes as well as cancer-related lncRNAs [12], showing superior discriminative performance in CPTAC dataset, although further validation in clinically heterogeneous and prospective cohorts will be required.

Overall, we report a somatic *TUSC7* mutation associated with altered transcript stability and stress-dependent functional effects. These findings highlight the strong diagnostic potential of *TUSC7* expression and support further investigation of its mutational status as a candidate biomarker in NSCLC.

## Electronic supplementary material

Below is the link to the electronic supplementary material.

Supplementary Material 1: **Additional File 1 – lncRNA_targets (.csv)**. BED-like table with the long non-coding RNA exon coordinates used in gene capture and targeted DNA sequencing. Overlapping lncRNAs are reported in separate rows.

Supplementary Material 2: **Additional File 2 – Sequencing metrics (.csv)**. Targeted-capture report summary for each sample. Sample_ID column indicates the sample identifier; TOTAL_READS column indicates the total number of sequenced reads; MEDIAN_TARGET_COVERAGE column indicates the median depth of coverage across target regions; PCT_EXC_DUPE column indicates the proportion of duplicate reads excluded (duplicate rate); PCT_SELECTED_BASES column indicates the proportion of reads mapping to target regions (on-target rate); FOLD_80_BASE_PENALTY column indicates the fold enrichment required for 80% of target bases to reach the mean coverage (a measure of coverage uniformity); PCT_TARGET_BASES_20X column indicates the proportion of target bases covered at ≥ 20× depth (callability metric).

Supplementary Material 3: **Additional File 3 – lncRNA_mutations (.csv)**. List of lncRNA-associated somatic mutations identified in NSCLC samples. Each row represents a single mutation observed in one sample. The columns contain hg38 genomic coordinates, reference and alternate alleles, allelic fractions, the affected lncRNAs, and metadata for the sample in which the mutation was identified.

Supplementary Material 4: **Additional File 4 – Functional_impact_scores (.csv)**. Predicted functional impact results. This file contains genomic variants characterized by their hg38 coordinates, reference and alternate alleles, and the affected lncRNAs. Functional impact metrics include CADD RawScore and PHRED scores, as well as the FATHMM-MKL non-coding score. The “Rank” column indicates each variant’s position in a prioritized list based on predicted functional impact. Missing values are present for indels and multiple nucleotide polymorphisms, as FATHMM-MKL only supports single nucleotide variants, and not all indels receive a score by CADD.

Supplementary Material 5: **Additional File 5 – Candidate_mutation_shortlist (.csv)**. Shortlist of 17 lncRNA mutations meeting the CADD PHRED>20 and FATHMM-MKL>0.98 filters. When a mutation affects different overlapping lncRNAs, these are separated by commas.

Supplementary Material 6: Supplemental Methods & Figures

## Data Availability

DNA-seq data discussed in this publication are accessible through the Sequence Read Archive under BioProject accession number PRJNA1303433. Data will be publicly available upon publication without time restriction. All other data and processing code are available from the corresponding author upon reasonable request.

## Abbreviations

NSCLC: Non-small cell lung cancer
lncRNA: Long non-coding RNA
SNV: Single nucleotide variant
cDNA: Complementary DNA
*TUSC7 WT*: *Wild-type TUSC7*
*TUSC7 MUT*: *Mutant TUSC7*
EV: Empty vector
gDNA: Genomic DNA
MFE: Minimum free energy
FPKM: Fragments per kilobase of transcript per million mapped reads
LUAD: Lung adenocarcinoma
LUSC: Lung squamous cell carcinoma
CI: Confidence interval
CPTAC: Clinical Proteomic Tumor Analysis Consortium
ROC: Receiver operating characteristic
AUC: Area under the curve
