# Supplementary material for: TUSC7 expression and mutational profile define its potential as a diagnostic and therapeutic biomarker in non-small cell lung cancer
Source: Biomark Res. 2026 Apr 10;14:39. doi: 10.1186/s40364-026-00916-0 (PMC13067388; doi:10.1186/s40364-026-00916-0)

TUSC7 expression and mutational profile define its potential as a diagnostic and therapeutic biomarker in non-small cell lung cancer

Martin-Lopez *et al.*

Supplemental Methods

Patient samples

DNA and RNA from 70 primary lung adenocarcinoma (LUAD) tumors and their matched adjacent normal tissues were obtained from the Basque Biobank and processed following standardized operating procedures, with appropriate approval from the relevant Ethical and Scientific Committees. Patients were diagnosed between August 2008 and January 2016. The cohort included 20 women (28.6%) and 50 men (71.4%), with 30 patients under 60 years of age (42.9%) and 40 over 60 (57.1%). The median age at diagnosis was 66.1 years (range 47.6–83.2), the median time to relapse was 17.4 months, and median overall survival was 20.1 months. All diagnoses were histologically confirmed by an experienced pathologist. The study was approved by the Research Ethics Committee of Granada (CEI Granada, Department of Health, Regional Government of Andalusia, Spain) and the Basque Foundation for Health Innovation and Research. All participants provided written informed consent in accordance with the Declaration of Helsinki and applicable institutional and national regulations.

Gene capture and targeted DNA sequencing

Gene capture and targeted DNA sequencing were performed on a panel of 39 NSCLC-derived cell lines, 70 primary lung adenocarcinoma tumors, and 27 matched adjacent normal tissues. The custom capture panel was designed using NimbleDesign software (Roche, v4.0) for hybridization with the NimbleGen SeqCap EZ Choice Library (Roche). It included 2481 exonic regions of 827 human long non-coding RNAs (lncRNAs) (Additional File 1), selected based on their putative involvement in cancer. Candidate lncRNAs were curated from multiple sources,

including LncRNADisease, lncRNAdb, the LncPath™ Human Cancer Array (System Biosciences), and scientific literature. Bait design was initially based on Ensembl v79 annotations and later updated to GENCODE v48 (Ensembl v114) for downstream analyses. Target coordinates were padded by ±200 nucleotides.

Library preparation was performed using the TruSeq DNA Sample Preparation Kit (Illumina). In brief, 300 ng of genomic DNA per sample were fragmented with a Covaris S2 sonicator (target size: 180–220 bp), followed by end repair, A-tailing, and ligation of Illumina adapters. After size selection and PCR amplification (9 cycles), libraries were hybridized to the biotinylated probes, and captured fragments were enriched using streptavidin-conjugated magnetic beads. A second round of PCR amplification (14 cycles) was performed post-capture. Final libraries were quality-controlled using NanoDrop (Thermo Scientific) and a Bioanalyzer 2100 system (Agilent). Paired-end sequencing (2 × 150 bp) was conducted on a NextSeq 500 system (Illumina) using the Mid Output Kit.

DNA-seq analysis and variant calling

Raw sequencing reads were first assessed for quality using FastQC v0.11.5, and adapter sequences were trimmed with Cutadapt. Reads were aligned to the hg38 reference genome using BWA-MEM v0.7.13. For somatic variant calling, GATK's Mutect2 v4.5.0.0 was applied using the 1000 Genomes panel of normals (August 2015 release) and gnomAD v4.1 as the germline resource. Twenty-seven paired tumor and non-tumoral adjacent tissue samples were processed in paired mode, whereas the remaining 43 patient tumor samples and 39 cell line samples were processed in tumor-only mode. Variants were filtered to retain only those with a PASS flag and a minimum allele fraction of 0.2. For unpaired samples, any variants present in the 27 non-tumoral adjacent tissue samples were additionally excluded. The filtered paired and unpaired VCF files were merged, and variants were annotated using ANNOVAR (2025Mar21). To further minimize potential germline variants, variants present in avsnp151, gnomAD v4.1, or 1000 Genomes were removed. Targeted DNA-sequencing quality-control metrics were calculated using PICARD and mosdepth tools.

Functional impact analysis

To assess the potential functional impact of the identified lncRNA variants, we used two complementary prediction tools: CADD and FATHMM-MKL. CADD (Combined Annotation–Dependent Depletion) integrates multiple genomic annotations to estimate the deleteriousness of variants, providing a PHRED-scaled score where, for example, 20 corresponds to the top ~1% most deleterious variants genome-wide. CADD v1.7 was run remotely via the official web server (https://cadd.gs.washington.edu/score) using the final VCF file as input. FATHMM-MKL is a computational method that predicts the functional impact of non-coding variants by combining sequence conservation and regulatory feature data through a multiple kernel learning framework. Higher scores indicate a higher likelihood of functional disruption. FATHMM-MKL was executed locally using the official Python script and pre-computed database available on GitHub (https://github.com/ThomasDOtto/fathmm). For compatibility, variant coordinates were converted from the hg38 to the hg19 genome assembly before running the analysis, and the resulting output coordinates were subsequently converted back to hg38 for downstream interpretation. Functional scores from CADD and FATHMM-MKL were merged, and a shortlist of 17 candidate mutations was obtained by applying thresholds of CADD PHRED > 20 and FATHMM-MKL non-coding score > 0.98. Further filtering for genes with at least 10 mutations in our cohort narrowed the list to two lncRNAs: *TUSC7* and *SOX2-OT*.

Secondary structure prediction

Secondary structure of both *TUSC7* wild-type (*TUSC7 WT*) and mutant *TUSC7* (*TUSC7 MUT*) was predicted using the RNAfold web server of the ViennaRNA WebSuite (Gruber et al., 2008). Predictions of RNA secondary structures were based on minimum free energy (MFE).

Genomic DNA/RNA extraction

From cell pellets, genomic DNA was purified using QuickExtract™ DNA Extraction Solution (Biosearch Technologies). Total RNA for real-time PCR was extracted using TRI Reagent (Sigma) following the manufacturer's guidelines.

DNAseq mutation validation and expression pattern of *TUSC7*

Patients harboring *TUSC7* mutations according to DNA-sequencing data were validated by Sanger sequencing. Mutations were validated both at genomic and cDNA levels. Genomic DNA and cDNA from patients were obtained as described above and used as template for a PCR reaction employing DreamTaq Green PCR 2X Master Mix (Cat#K1081, ThermoFisher Scientific). The PCR products were purified from 1.5% agarose gels (Cat#740609.250, NucleoSpin® Gel and PCR Clean-up, Macherey-Nagel) following the manufacturer's protocol and sent for Sanger sequencing (StabVida, Setubal, Portugal). The following oligonucleotides were used: TUSC7 exon 1 forward, 5'-CTACTCCCTCTCTGCAAAGGC-3; TUSC7 exon 1 reverse, 5'-CTCCTCTCCCACATCTTCATCT-3'; TUSC7 forward, 5'-TCCTTGTGGAGGCCAAACTG-3'; TUSC7 reverse, 5'-GCTGTCAGAGCAGTCACAC-3'.

Cell culture

Non-small cell lung carcinoma (NSCLC) cell lines were cultured under standard conditions (37 °C, 5% CO_2) using either DMEM or RPMI 1640 medium according to individual cell line requirements. All media were supplemented with 10% fetal bovine serum (FBS) (Cat#10270-106, GibcoTM, ThermoFisher Scientific), 1% L-glutamine (Cat#X0550-100, Biowest, Riverside, MO, USA), 1% amphotericin (Cat#L0009-100, Biowest), and 100 U/ml streptomycin/penicillin (Cat#P0781-100ML, Sigma-Aldrich). RPMI 1640 was used for most lines, including LC319, LXF289, PC14, PC9, H1373, H1395, H1437, H1568, H1573, H1623, H1650, H1734, H1792, H1944, H1975, H2030, H2087, H2122, H2228, H23, H322, H358, H441, H522, H838, HCC827, HCC4006, HCC44, NCI-H460, NCI-H1299, and H1703. DMEM was used for A427, A549, CALU3, H1435, H1648, H1793, H2009, H2126,

H650, SK-MES-1 and SKLU1, according to standard growth conditions. AALE cells were cultured in SABM medium (Cat#CC-3119, Lonza).

Plasmids

HIV packaging (psPAX2) and VSV-G (pMD2.G) plasmids were acquired from Addgene (Addgene plasmids #12260 and #12259 respectively). The psPAX2 plasmid encodes for the packaging genes gag, pol, tat and rev. The pMD.G plasmid encodes the vesicular stomatitis virus G protein. The lentiviral vectors containing a stuffer (empty vector)(pLV[Exp]-EGFP:T2A:Puro-EF1A>ORF_Stuffer), a wild-type (pLV[ncRNA]-EGFP:T2A:Puro-CMV >hTUSC7 [NR_015391.1]) or a mutant version (pLV[ncRNA]-mCherry:T2A:Puro-CMV>{TUSC7 mut exon 4}) of *TUSC7* were constructed by VectorBuilder. The mutant *TUSC7* construct (*TUSC7 MUT*) includes a somatic variant identified in a tumoral sample: chr3:116716439 T>A (GRCh38/hg38), corresponding to c.1510T>A in the transcript NR_015391.1). This mutation, located in exon 4, was validated by Sanger sequencing in both genomic DNA and cDNA, and was selected based on its predicted impact on RNA secondary structure and its exclusive presence in tumoral tissue.

Lentiviral production, titration and cell line transduction

Lentiviral production was carried out in HEK293T cells by lipofection. Packaging cells were grown in DMEM supplemented with 10% fetal bovine serum (FBS), 100 U/ml streptomycin and penicillin. The day before transfection, cells were plated in a 100-mm tissue culture grade Petri dish (Cat#734-2321, VWR European). Lentiviral, packaging (psPAX2) and envelope (pMD2.G) plasmids (7.5, 5 and 2.5 μg respectively) were resuspended in 0.5 ml Opti-MEM reduced serum medium (Cat# 31985047, Gibco™, ThermoFisher Scientific) and mixed with 45 μl LipoD293 (Cat#SL100668, Signagen Laboratories, Rockville, MD, USA) previously diluted in 0.5 ml Opti-MEM reduced serum medium during 20 min at room temperature. The plasmid – lipoD293 complexes were added to cells. Five hours after transfection, transfection mixture was removed and 7 ml of complete medium were added. Viral supernatants were collected, filtered through a 0.45 μm filter (Cat#514-1261, VWR

European), aliquoted and stored at -80ºC. Viral titers (transduction units/ml) were calculated based on the percentage of fluorescence$^+$ cells (EGFP for empty vector and *TUSC7* wild-type, mCherry for mutant *TUSC7*) detected in the linear range of a serial dilution of viral supernatant using a highly permissive cell line such as K-562. The H460 cell line was transduced with either pLV[ncRNA]-EGFP:T2A:Puro-CMV >hTUSC7 [NR_015391.1], pLV[ncRNA]-mCherry:T2A:Puro-CMV>{TUSC7 mut exon 4} or an empty vector for control. Cells were transduced at MOI=3. H1299 and SK-MES-1 cell lines were transduced with either pLV[ncRNA]-EGFP:T2A:Puro-CMV >hTUSC7 [NR_015391.1] or pLV[ncRNA]-mCherry:T2A:Puro-CMV>{TUSC7 mut exon 4}. Cells were transduced at MOI=3. Transduced mCherry$^+$ or EGFP$^+$ cells were FACS sorted.

Colony formation assay

For the colony formation assay, 1000 sorted cells per condition were seeded into 6 well plates. After 4 days in culture, cells were incubated at room temperature with 0.1% of crystal violet, 1% methanol, 1% formaldehyde solution for 20 minutes to fix and stain colonies. Plates were washed with water, images of colonies were acquired, and colonies were quantified using a LI-COR Odyssey system. Three biological replicates were performed for each condition.

Competitive assay

For the H460 competition assay under normal conditions, both pLV[ncRNA]-EGFP:T2A:Puro-CMV>hTUSC7 [NR_015391.1] and pLV[ncRNA]-mCherry:T2A:Puro-CMV>{TUSC7 mut exon 4} were mixed at a 1:1 ratio. Sorted cells were tracked by measuring the abundance of EGFP$^+$ or mCherry$^+$ populations over time. Measures were taken at the start (day 0), middle (day 4), and the end of the experiment (day 7), and growth defects were monitored by fluorescence-activated cell sorting (FACS) analysis (BD FACSVerse™, BD Biosciences, San Jose, CA, USA). The percentage of EGFP$^+$ or mCherry$^+$ at day 4 and 7 was normalized to the percentage of EGFP$^+$ or mCherry$^+$ at day 0. For the competition assay under genotoxic conditions, the sorted cells were mixed with non-transduced cells (1:1 ratio). Sorted cells were tracked by

measuring the abundance of EGFP$^+$ or mCherry$^+$ populations over time. Measures were taken at the start (day 0), middle (day 4), and the end of the experiment (day 7), and growth defects were monitored by fluorescence-activated cell sorting (FACS) analysis over time (BD FACSVerse™, BD Biosciences, San Jose, CA, USA). The percentage of EGFP$^+$ or mCherry$^+$ at day 4 and 7 was normalized to the percentage at day 0 and subsequently normalized to the empty vector. Three biological replicates were performed for both basal and genotoxic stress conditions. For the SK-MES-1 and H1299 competition assays under both normal and genotoxic conditions, the sorted cells were mixed with non-transduced cells (1:1 ratio). Sorted cells were tracked by measuring the abundance of EGFP$^+$ or mCherry$^+$ populations over time. Measures were taken at the start (day 0) and the end of the experiment (day 7), and growth defects were monitored by fluorescence-activated cell sorting (FACS) analysis over time (BD FACSVerse™, BD Biosciences, San Jose, CA, USA). The percentage of EGFP$^+$ or mCherry$^+$ on day 7 was normalized to the percentage on day 0. Three biological replicates were performed for both basal and genotoxic stress conditions. Competitive assays under genotoxic conditions were performed by supplementing the medium with doxorubicin hydrochloride (0.2 µg/ml) for 4 days.

Cell viability assays

500 sorted cells per condition (H460 *TUSC7* wild-type or *TUSC7* mutant) were seeded into 96-well plates, with three technical replicates per condition. Cell viability was measured at 0, 3, and 5 days under basal conditions and at 0, 3, 5, and 7 days under genotoxic conditions by adding resazurin sodium salt solution (0.12 mM) (Sigma-Aldrich, Merck) and incubating in darkness at 37 °C for 4 hours. Then, 3% SDS was added and fluorescence at 600 nm was measured using a GloMax® Discover Multimode Microplate Reader. Three and two biological replicates were performed for assays under basal and genotoxic stress conditions, respectively. Viability assays under genotoxic stress were performed by supplementing the medium with doxorubicin hydrochloride (0.05 µg/ml) throughout the assay.

Cell proliferation assays

5 × 10^5 cells were labeled with the CellTrace™ Violet Proliferation Kit (Cat# C34557, Thermo Fisher Scientific) according to the manufacturer's instructions. Proliferation was assessed at days 0, 2, and 4 by flow cytometry using a BD FACSVerse™ cytometer (BD Biosciences, San Jose, CA, USA), measuring CellTrace™ Violet fluorescence intensity. Median fluorescence intensity (MFI) was determined for each time point. Proliferation curves were generated using the inverse of the normalized median fluorescence intensity values (1/MFI) and fitted to an exponential growth model, from which doubling times were calculated for each experimental condition. For proliferation assays under genotoxic stress, cells were cultured in the presence of 0.2 µg/mL doxorubicin (Doxorubicin Aurovitas, 2 mg/mL stock solution), and analyses were performed as described above. Three and four independent biological replicates were performed for basal and genotoxic stress conditions, respectively.

Cell migration assays

For the migration assays, sorted cells were cultured in 6-well plates until 90% confluence. Cells were then maintained in serum-reduced medium containing 1% FBS, and wounds were created using a pipette tip. Three technical replicates were performed for each condition. Wound closure was monitored for 48 hours, with images acquired at 0, 24, and 48 hours. For the migration assays under genotoxic conditions, after seeding, cells were incubated with complete medium containing mitomycin (5 µg/ml) for 2 hours. Cells were then refreshed with medium, and wounds were created and monitored for 120 hours, with images acquired at 0, 24, 48, 72, and 120 hours. Wound area and width were measured using ImageJ software applying the plugin "Wound Healing Size Tool". Two biological replicates were performed under basal and genotoxic conditions.

Cisplatin dose-response curves

Sorted SK-MES-1 *TUSC7* wild-type (*WT*) or mutant (*MUT*) cells were plated at 1,000 cells per well. The same day, cells were treated with cisplatin (starting

concentration, 100 μM; 15-point dose titration; 1:2 serial dilutions). After 4 days, cell viability was measured by adding resazurin sodium salt solution (0.12 mM) (Sigma-Aldrich, Merck, #199303) and incubating in darkness at 37 °C for 4 hours. Then, 3% SDS was added and fluorescence at 660 nm was measured in a GloMax® Discover Multimode Microplate Reader. Viability was calculated relative to DMSO-treated cells (100% viability). IC_{50} values were calculated from the resulting viability curves. Four biological replicates were performed.

Actinomycin D mRNA stability assays

To assess mRNA stability, 5×10^5 sorted H460 *TUSC7 WT* and *TUSC7 MUT* cells were treated with actinomycin D (5 μg/mL) for the indicated time points (0, 60, and 120 min). At each time point, *TUSC7* mRNA levels were quantified by RT–qPCR and normalized to *GAPDH* expression. *c-MYC* mRNA levels were measured as a positive control for transcript decay.

Experiments were performed under both basal and genotoxic stress conditions. For genotoxic stress, cells were cultured in medium supplemented with 0.2 μg/mL doxorubicin (Doxorubicin Aurovitas, 2 mg/mL stock solution). Three independent biological replicates were performed for both basal and genotoxic stress conditions.

Reverse transcription quantitative PCR (RT-qPCR)

2 μg of RNA were treated with DNase I (Invitrogen) and reverse transcribed using the RevertAid RT kit (Thermo Fisher Scientific) to remove contaminating DNA and synthesize complementary DNA (cDNA), respectively. cDNA was used as template for SYBR Green-based quantitative PCR to quantify gene expression using KAPA SYBR® FAST (Merck) and the QuantStudio™ 3 Real-Time PCR System (Thermo Fisher Scientific). Three technical replicates were performed for each biological replicate. The ΔΔCt method was used to calculate relative expression, normalized to *GAPDH* expression. The following oligonucleotides were used: TUSC7 forward, 5'-TCCTTGTGGAGGCCAAACTG-3'; TUSC7 reverse, 5'-GCTGTCAGAGCAGTCACACTT-3'; GAPDH forward, 5'-GAAGGTGAAGGTCGGAGTC-3'; GAPDH reverse, 5'-GAAGATGGTGATGGGATTTC-3'; c-MYC forward, 5'-CCACCAGCAGCGACTCTGAG-

3'; c-MYC reverse, 5'-CCAGCAGAAGGTGATCCAGAC-3'. Expression levels of *TUSC7* wild-type and mutant constructs were verified post-transduction (three biological replicates) and confirmed to be comparable across conditions, ensuring that observed phenotypic effects were not attributable to differential expression.

CPTAC data analysis

Data used in this publication were generated by the Clinical Proteomic Tumor Analysis Consortium (NCI/NIH). Publicly available RNA expression data from paired tumor and matched adjacent non-tumoral tissue samples of lung adenocarcinoma (LUAD; 215 tumor and 215 matched normal samples) and lung squamous cell carcinoma (LUSC; 97 tumor and 96 matched normal samples) were downloaded via the Genomic Data Commons (GDC) portal. For the single LUSC patient with two tumor samples, the FPKM values were averaged prior to downstream analysis.

Receiver Operating Characteristic (ROC) curve analysis

To evaluate the discriminative performance of gene expression levels between tumor and matched normal tissue samples, Receiver Operating Characteristic (ROC) curve analyses were performed. An adjusted analysis was conducted using a generalized linear mixed-effects model (GLMM), including tumor status as the binary outcome, *TUSC7* expression as the predictor, and patient ID as a random intercept to account for intra-patient correlation. Model-derived predicted probabilities were used to construct ROC curves, and area under the curve (AUC) values were calculated. The area under the ROC curve (AUC) was calculated as a measure of classification performance, with 95% confidence intervals estimated using non-parametric bootstrapping. All statistical analyses were performed in R using the pROC package for ROC analysis and the lme4 package for mixed-effects modeling.

Statistical analysis

Statistical analysis was performed using GraphPad Prism. Biological replicates refer to independently cultured and treated cell populations processed on separate days. For all analyses, p-values ≤ 0.05 were considered statistically significant.

For NCI-H460 competitive assays, differences across time points and conditions were analyzed using two-way ANOVA followed by Tukey's multiple comparisons test. For NCI-H1299 and SK-MES-1 models, comparisons between *TUSC7 WT* and *MUT* variants under basal and genotoxic conditions were assessed using unpaired two-sided t-tests at day 7 of the competitive assay. Differences in colony formation were analyzed using one-way ANOVA with Tukey's multiple comparisons test. Cell viability differences were evaluated using two-way ANOVA with Tukey's multiple comparisons test. Migration assays were analyzed using one-way ANOVA with Tukey's multiple comparisons test.

For proliferation assays, statistical differences across time points (day 0, 2, and 4) were analyzed using one-way ANOVA with Tukey's multiple comparisons test. Doubling times were calculated using an exponential growth model, and differences between doubling times were assessed using one-way ANOVA with Tukey's correction. In cisplatin dose-response experiments, IC_{50} values were calculated using non-linear regression models, and differences between IC_{50} values were evaluated using an unpaired two-sided t-test. For actinomycin D experiments, statistical analysis was performed using two-way ANOVA with Sidak's multiple comparisons test. For patient-derived *TUSC7* expression analyses, because tumor and normal samples were paired within each patient, differences were assessed using a paired two-sided Wilcoxon signed-rank test. All quantitative data are represented as mean ± standard deviation (SD).

Supplemental Figures

Supp. Figure 1. H460 lacks *TUSC7* expression. **(a)** *TUSC7* mRNA expression levels in several NSCLC cell lines, including the immortalized tracheobronchial epithelial AALE cell line as a healthy control. *TUSC7* expression is presented as the base-2 logarithm of expression levels, normalized to H460 expression. **(b)** PCR amplification of exon 1 and exon 4 of *TUSC7* in genomic DNA from H460 and A549 cell lines. GAPDH and non-DNA controls were included.

Supp. Figure 2. *TUSC7* variants overexpression in NSCLC models. **(a)** Representative flow cytometry plots showing H460 cell line transduction efficiency for both *TUSC7* mutant (*MUT*) and *TUSC7* wild-type (*WT*), assessed by measuring mCherry (upper panels) and EGFP (bottom panels) levels, respectively. A non-transduced cell line was employed as a negative control. **(b)** Log_2 *TUSC7* mRNA expression levels in the H460 cell line transduced with either *TUSC7 WT* or *MUT*. The cell line transduced with an empty vector (EV) was used as a control. **(c)** Representative flow cytometry plots showing SK-MES-1 cell line transduction efficiency for both *TUSC7 MUT* and *TUSC7 WT*, assessed by measuring mCherry (upper panels) and EGFP (bottom panels) levels, respectively. A non-transduced cell line was employed as a negative control. **(d)** Log_2 *TUSC7* mRNA expression levels in the SK-MES-1 cell line transduced with either *TUSC7 WT* or *MUT*. The non-transduced cell line was employed as a negative control. **(e)** Representative flow cytometry plots showing H1299 cell line transduction efficiency for both *TUSC7 MUT* and *TUSC7 WT*, assessed by measuring mCherry (upper panels) and EGFP (bottom panels) levels, respectively. A non-transduced cell line was employed as a negative control. **(f)** Log_2 *TUSC7* mRNA expression levels in the H1299 cell line transduced with either *TUSC7 WT* or *MUT*. The non-transduced cell line was employed as a negative control.

Supp. Figure 3. Mutated *TUSC7* functional assays. **(a)** Competitive assay in the H460 cell line after transduction with lentiviral particles carrying wild-type (*WT*) *TUSC7* or mutant (*MUT*) *TUSC7*. The different conditions were normalized to day 0. Three biological replicates were performed. **(b)** Quantification of clonogenic assays

performed under normal conditions on the H460 cell line transduced with EV, *TUSC7 WT*, or *TUSC7 MUT*. Three biological replicates were performed. **(c)** Viability assays performed in normal (upper) or genotoxic (bottom) conditions on the H460 cell line transduced with EV, *TUSC7 WT*, or *TUSC7 MUT*. Growth was normalized to day 0. Three and two biological replicates were performed under basal and genotoxic stress conditions, respectively. (168 h H460 EV vs H460 *TUSC7 MUT*, p = 0.002, mean diff: -0.35 (95% CI -0.56 – -0.14)). **(d)** Proliferation assays in the H460 cell line transduced with EV, *TUSC7 WT*, or *TUSC7 MUT* under basal (left panel, Doxo−) and genotoxic stress (right panel, Doxo+) conditions. CellTrace signal indicates median fluorescence intensity (MFI) normalized to day 0 for each time point. Three and four biological replicates were performed under basal and genotoxic stress conditions, respectively. **(e)** Representative growth curves (left panel) of the H460 cell line transduced with EV, *TUSC7 WT*, or *TUSC7 MUT* under both basal (Doxo−) and genotoxic stress (Doxo+) conditions. Proliferation curves were expressed as 1/CellTrace signal for each time point and fitted to an exponential growth model from which doubling times were calculated (right panel). CellTrace signal indicates median fluorescence intensity (MFI) normalized to day 0 for each time point. ns: not significant. **(f)** Wound healing assays performed in normal (left) or genotoxic (right) conditions on the H460 cell line transduced with EV, *TUSC7* WT, or *TUSC7* MUT. Wound closure (%) and cell migration rate were normalized to EV. Two biological replicates were performed. ns: not significant; * $p < 0.05$; ** $p < 0.01$.

Supp. Figure 4. Uncropped gel images showing PCR amplification of exon 1 and exon 4 of TUSC7 in genomic DNA from H460 and A549 cell lines. GAPDH and non-DNA controls were included.

Supplementary Figure 1

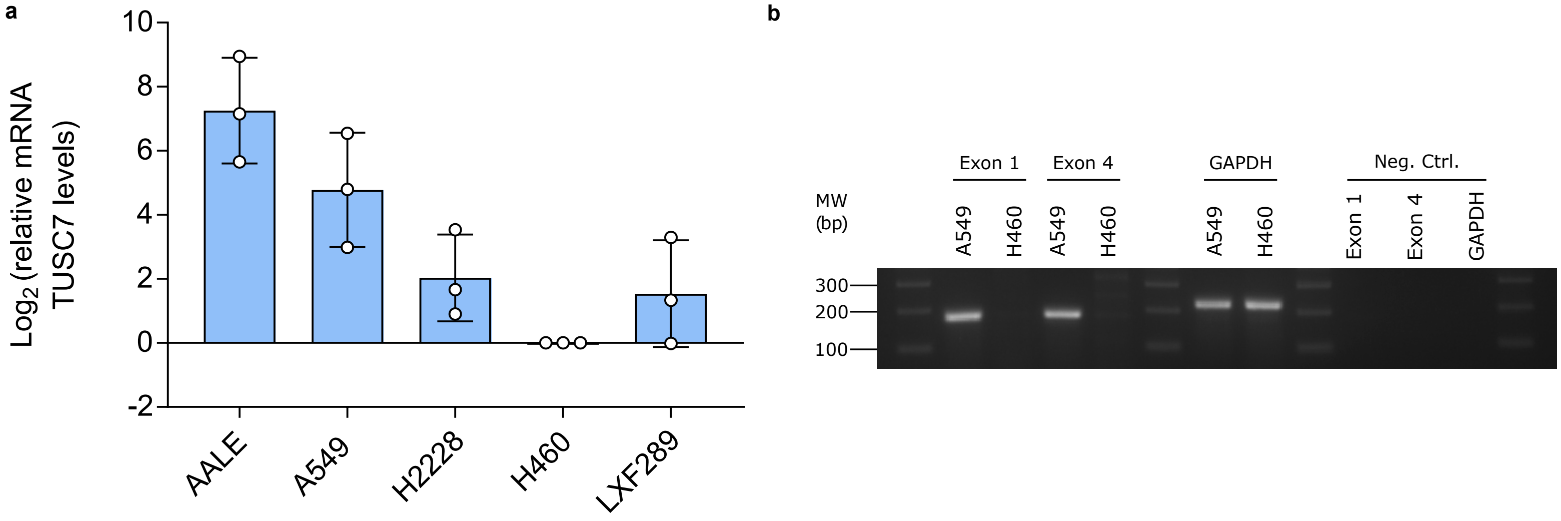

Supplementary Figure 2

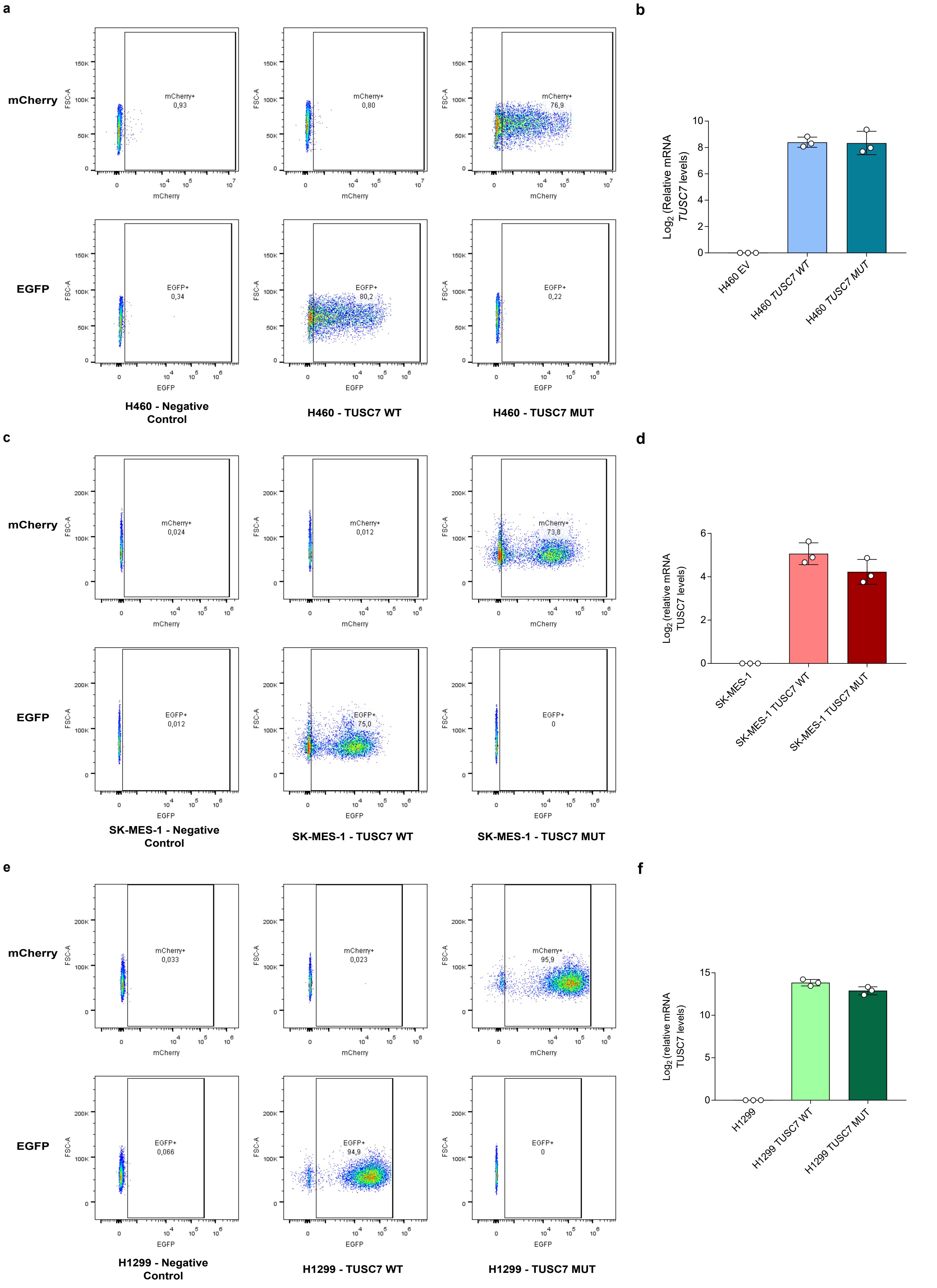

Supplementary Figure 3

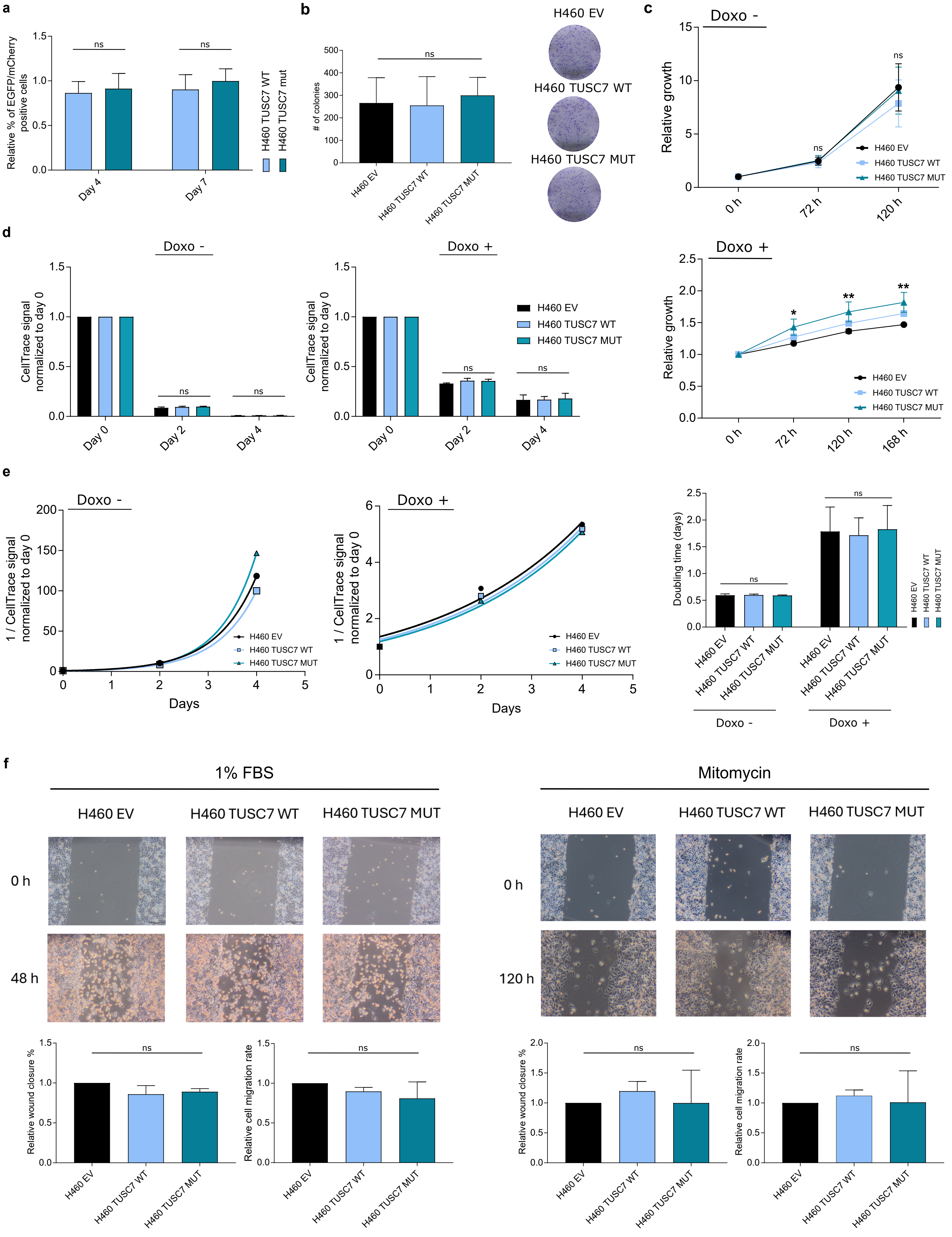

Supplementary Figure 4

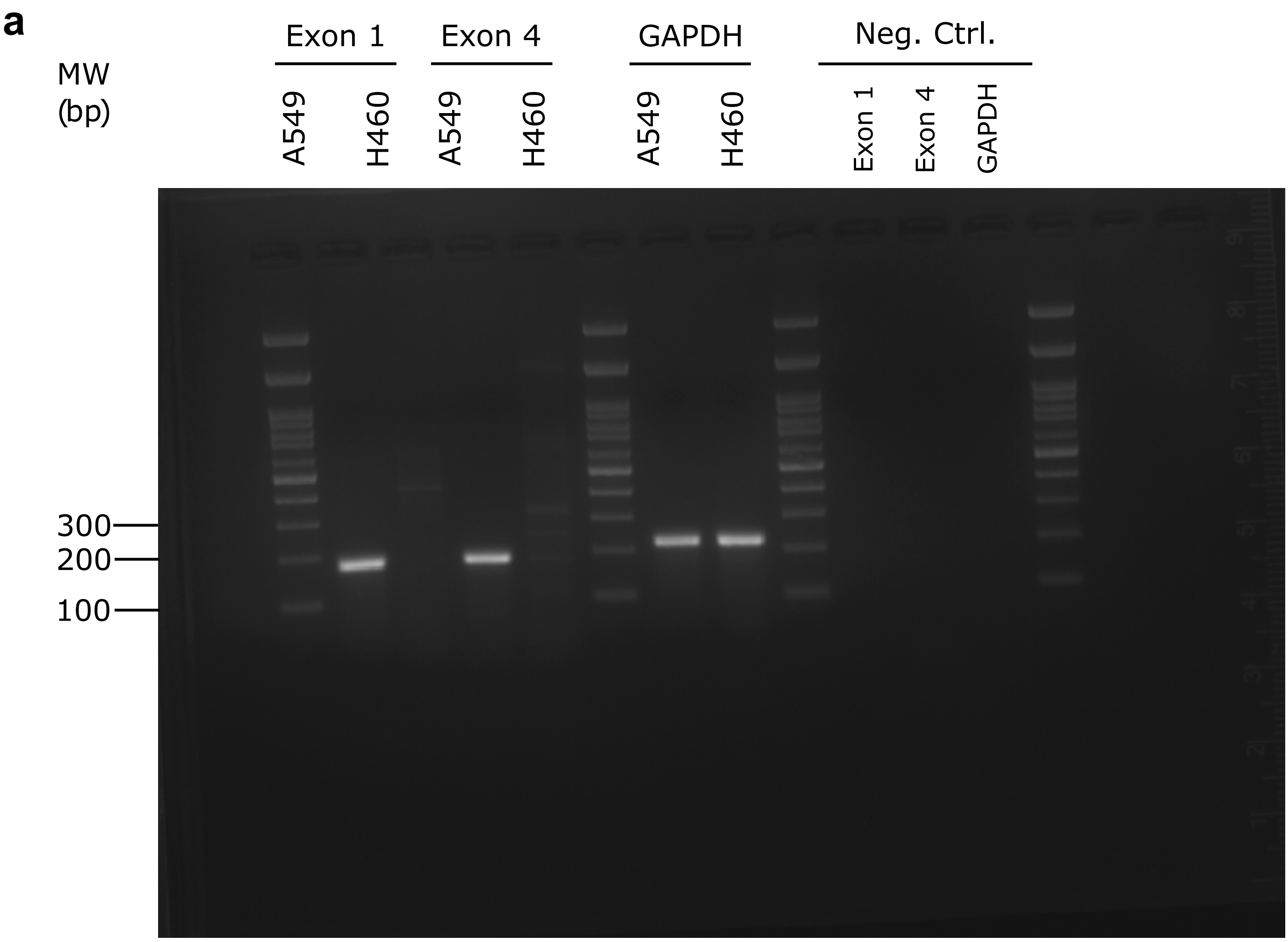

Supplement: Supplementary file 6 — Supplementary Material 6: Supplemental Methods & Figures [file 40364_2026_916_MOESM6_ESM.pdf]
